# H7N9 Avian Influenza Virus Is Efficiently Transmissible and Induces an Antibody Response in Chickens

**DOI:** 10.3389/fimmu.2018.00789

**Published:** 2018-04-13

**Authors:** Peirong Jiao, Yafen Song, Jianni Huang, Chengwei Xiang, Jin Cui, Siyu Wu, Nannan Qu, Nianchen Wang, Guowen Ouyang, Ming Liao

**Affiliations:** ^1^College of Veterinary Medicine, South China Agricultural University, Guangzhou, China; ^2^China Institute of Veterinary Drug Control, Beijing, China; ^3^China Animal Health and Epidemiology Center, Qingdao, China

**Keywords:** H7N9, avian influenza virus, pathogenicity, transmission, immune response

## Abstract

H7N9 viruses pose a threat to human health and they are no less harmful to the poultry industry than the H5N1 avian influenza viruses. However, the pathogenesis, transmissibility, and the host immune response of the H7N9 virus in chickens and mice remain unclear. In this study, we found that H7N9 viruses replicated in multiple organs of the chicken and viral shedding persisted up to 30 days postinoculation (DPI). The viruses were efficiently transmitted between chickens through direct contact. Notably, chickens infected with H7N9 had high antibody levels throughout the entire observation period and their antibody response lasted for 30 DPI. The expression levels of the pattern-recognition receptors and pro-inflammatory cytokines were found to be significantly upregulated in the brain using quantitative real-time PCR. The expression of TLR3, TLR7, MDA5, Mx, IL-1β, IL-6, IFN-α, and IFN-γ were also significantly different in the lungs of infected chickens. We found that the viruses isolated from these birds had low pathogenicity in mice, produced little weight loss and could only replicate in the lungs. Our findings suggested that the H7N9 viruses could replicate in chickens and mice and be efficiently transmitted between chickens, which presented a significant threat to human and poultry health.

## Introduction

Avian influenza is a zoonosis caused by the avian influenza virus, which can infect birds and mammals. Based on its effects in chickens, avian influenza viruses can be divided into highly pathogenic avian influenza (HPAI) viruses and low pathogenic avian influenza (LPAI) viruses. LPAI viruses usually cause a much milder, primarily respiratory disease. HPAI viruses are generally restricted to subtypes of H5 and H7 and cause mild to severe illnesses with high mortality (1).

In March 2013, the H7N9 LPAI virus first caused a human death in Shanghai, China (2). As of November 21, 2016, 800 cases of human infection with the H7N9 influenza virus had been confirmed with 322 fatalities (3). This is the first time that human infection with an LPAI virus has been associated with a high mortality rate. The total number of H7N9 infections in a 4-year period was similar to the case count for H5N1 over the past two decades (4, 5). In addition, the five epidemic waves of H7N9 influenza have led to the spread of this virus across 33 Chinese provinces (6). Moreover, compared to H5N1 viruses, H7N9 viruses spread silently among poultry and can be very difficult to find due to the lack of symptoms in birds (7). However, the H7N9 LPAI virus can be detected from poultry in live-bird markets. Therefore, H7N9 virus represents a serious threat to public health and could cause considerable economic losses in the poultry industry.

The pathogenicity of the avian influenza virus is multifactorial. Both virulence and host factors contribute to the pathogenesis of susceptible chickens (8). Most avian influenza virus crossover events into humans have occurred through close contact with domestic poultry (9). Previous studies have shown that the majority of human H7N9 infections have resulted from exposure to live-poultry markets (10). However, to date, the pathogenicity and transmission mechanisms of H7N9 infection in chickens have not been characterized. Although chickens are considered to be the primary source of H7N9 infection in humans, chickens infected with the H7N9 virus show no clinical signs or mortality. Chickens can be infected with the H7N9 virus isolated from humans and shed the virus for up to 14 days without showing any clinical symptoms, but the virus can still be successfully transmitted through direct contact (11, 12). However, other studies have found that the H7N9 virus, whether isolated from poultry or humans, could not replicate efficiently in chickens and be transmitted poorly through direct contact (13, 14).

The pathogenicity of the avian influenza virus is influenced by the host’s response. Serological studies typically provide values for the true incidence or prevalence of a suspected pathogen, infection with a pathogen, and immune protection from pathogen (15, 16). Previous studies have demonstrated that the high morbidity and mortality of human infections with HPAI viruses are usually associated with aberrant cytokine production. Hypercytokinemia and high viral loads play an important role in the disease progression and the ultimate death of patients infected with the H5N1 avian influenza virus (17). However, the role of the immune response and cytokines in the pathogenic mechanism of H7N9 infection in chickens remains unknown. We investigated the pathogenicity, transmission, and immune response of the H7N9 avian influenza viruses in chickens and mice, isolated from cloacal swabs in live-poultry markets in Guangdong, China, in 2013.

## Materials and Methods

### Sample Collection

In 2013, swab samples were collected from healthy chickens in live-poultry markets in Guangdong, Southern China. This was performed using plain-flocked swabs that were individually wrapped in a plain dry tube (Thermo Fisher Scientific, China. Cat. No. 552C). Before use, the plain-flocked swabs were placed in 2 ml of isotonic phosphate-buffered saline (PBS, pH = 7.4) supplemented with penicillin (2,000 U/ml) and streptomycin (2,000 U/ml). The samples were transported at low-temperatures (−25–10°C) and stored at −80°C prior to use.

### Viruses Isolation

The viruses were isolated in accordance with standard procedures (18). Briefly, the supernatant from cloacal samples was obtained through clarification by centrifugation at 1,000 × *g* and inoculated into the allantoic cavity of five 9–10-day-old specific-pathogen-free (SPF) embryonated hen eggs at 200 µl of swab material per egg. The eggs were incubated at 37°C for 72 h. The allantoic fluids were collected and stored at −80°C until used.

Two H7N9 avian influenza viruses, A/chicken/Guangdong/110/2013 (CK110) and A/chicken/Guangdong/134/2013 (CK134), were identified using reverse transcription polymerase chain reaction (RT-PCR), hemagglutination tests, and hemagglutination inhibition (HI) tests as standard protocols. Detailed information describing these methods is available in previous publications (19). Evaluation of 50% egg infective doses (EID_50_) was calculated using the Reed–Muench method (20). All experiments were carried out in facilities with Animal Biosafety Level 3 (ABSL-3) at South China Agricultural University.

### Infection Studies in Chickens

Six-week-old SPF White Leghorn chickens were purchased from Guangdong Wens Dahuanong Biotechnology Co., Ltd. 22 chickens were randomly divided into two groups of 11 chickens each. The chickens in each group were inoculated intranasally with10^8^ EID_50_ of CK110 and CK134 viruses in a 0.2 ml volume, respectively. Five chickens were placed in each group to allow for contact with the inoculated chickens for 24 h postinoculation. To enable individual identification, each chicken was numbered with a metal ring to the leg. At 3 and 5 days postinoculation (DPI), three chickens of each inoculated group were euthanized. And the brain, spleen, kidneys, lungs, liver, intestines, heart, trachea, and pancreas of them were collected to detect the virus, respectively. The remaining chickens were observed for clinical symptoms and monitored for viral shedding. We also investigated the pattern-recognition receptors (PRRs) and cytokines in the lungs and brains, which were collected from three euthanized chickens of CK134-inoculated group at 3 DPI.

Oropharyngeal and cloacal swab samples from the chickens in the CK110-inoculated group and CK110-contacted group were collected at 3, 5, 7, 9, 11, and 14 DPI. In order to determine how long viral shedding and elevated antibody levels persisted, the CK134-inoculated group and CK134-contacted group were monitored for 30 days. Swab samples from chickens in the CK134-inoculated group and CK134-contacted group were collected at 3, 5, 7, 9, 11, 14, 17, 20, 23, 25, 28, and 30 DPI, and blood samples were collected at 9, 11, 14, 17, 21, 25, 28, and 30 DPI (21).

In addition, 11 chickens were not given any treatment as a control group. Three control chickens were euthanized at three DPI and their tissues were assayed as described above. The remaining chickens from the control group were observed for clinical symptoms for 14 days.

The collected samples (1 g per tissue) were homogenized in 1 ml of PBS supplemented with penicillin (1,000 U/ml) and streptomycin (1,000 U/ml) and were centrifuged at 4,000 × *g* to isolate supernatant fluids. The resulting supernatants were serially diluted by a factor of 10 and inoculated into the allantoic cavity of 9–10-day-old embryonated eggs (100 µl per egg). The eggs were incubated at 37°C for 48 h. The virus titers were detected by the HA test and calculated using the method of Reed and Muench method (20).

The swab samples were suspended in 1 ml of PBS and inoculated into the 9–10-day-old SPF eggs. At the end of the incubation period, the allantoic fluids were collected and tested for HA activity with 1% (v/v) chicken red blood cells. When the HA assay was positive, the allantoic fluids were used to extract the viral RNA. Toward determining the viral presence, RT-PCR was performed using primers designed for viral detection and the HA gene of the virus was then sequenced.

### Quantification of PRRs and Cytokines in Chicken Lung and Brain Tissue

To determine the immune response of chickens inoculated with the H7N9 virus, total RNA was extracted from the lung and brain tissue of three euthanized chickens from the CK134-inoculated group and control group using the RNeasy Plus Mini Kit (Qiagen, Germany) according to the manufacturer’s instructions. Total RNA (1 µg) was reverse transcribed with the SuperScript III First Strand Synthesis System (Invitrogen, China). Real-time quantitation of the mRNA relative to β-action was performed with a SYBR Green I assay (Qiagen, Germany). The primers used for qRT-PCR were designed using the Oligo 7 software (Molecular Biology Insights Inc., Cascade, CO, USA).

Primer pairs were selected based on specificity determined by dissociation curves. The qRT-PCR was carried out using a 7500 Fast Real-Time PCR system (Applied Biosystems, Foster City, CA, USA). The PCR conditions were as follows: 1 cycle of 95°C for 5 min, followed by 40 cycles of 95°C for 15 s and 60°C for 34 s. The increase in fluorescence was measured in real time during the extension step. The cycle threshold (CT) was determined and then the relative gene expression was expressed as follows: fold change = 2^–ΔΔ Ct,^ where Δ Ct = Ct target − Ct β-action, and ΔΔ Ct = ΔCt treated − ΔCt control.

### Infection Studies in Mice

Five-week-old female SPF BALB/c mice were purchased from the Guangdong Medical Laboratory Animal Center. To evaluate the pathogenicity of the H7N9 viruses in mice, 22 mice were randomly divided into two groups of 11 mice each. In each group, the mice were anesthetized using CO_2_ and inoculated intranasally with CK110 and CK134 viruses at doses of 50 µl of viral stock, respectively.

Three mice from each inoculated group were euthanized at 3 and 5 DPI and their brain, spleen, kidneys, and lungs were collected for further analysis. Additionally, 11 mice inoculated with 50 µl of PBS served as negative controls. Three control mice were euthanized at 3 and 5 DPI, and the same organs were assayed. The remaining in all three groups were checked daily for weight loss, signs of disease, and mortality for 14 DPI (21). All of the tissues were ground and homogenized in 1 ml of PBS, the homogenates were pelleted by centrifugation at 4,000 × g for 5 min, and the supernatants were used to determine for viral titration in 9–10-day-old SPF embryonated hen eggs (22).

### Ethics Statement

All experiments were carried out in ABSL-3 facilities in compliance with approved protocols (SCAUABSL2015-0011) by the biosafety committee of South China Agriculture University. All animals were handled in accordance with the approved guidelines of the Experimental Animal Administration and Ethics Committee of South China Agriculture University approved guideline.

### Statistical Analysis

**Figure 1:**
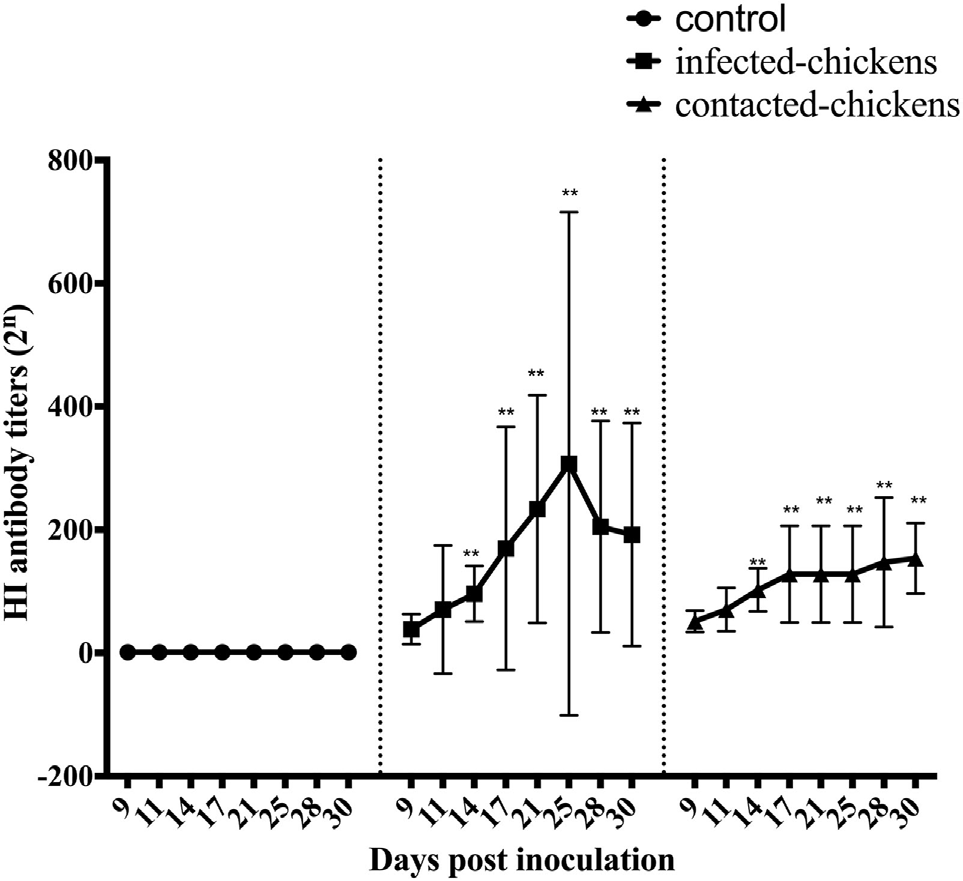
The hemagglutination inhibition (HI) antibody titers of infected and contacted chickens. Serum samples were collected from CK134-inoculated chickens and naïve chickens in the same isolator, and all of the samples were analyzed by HI tests. An HI titer >2^3^ was considered indicative of the CK134 virus, whereas titers <2^3^ were considered negative (12). Statistical analysis was performed using the two-way ANOVA test (GraphPad Prism 7.0 software), *p* < 0.05 was considered statistically significant (*p < 0.05; **p < 0.01).

**Figure 2:**
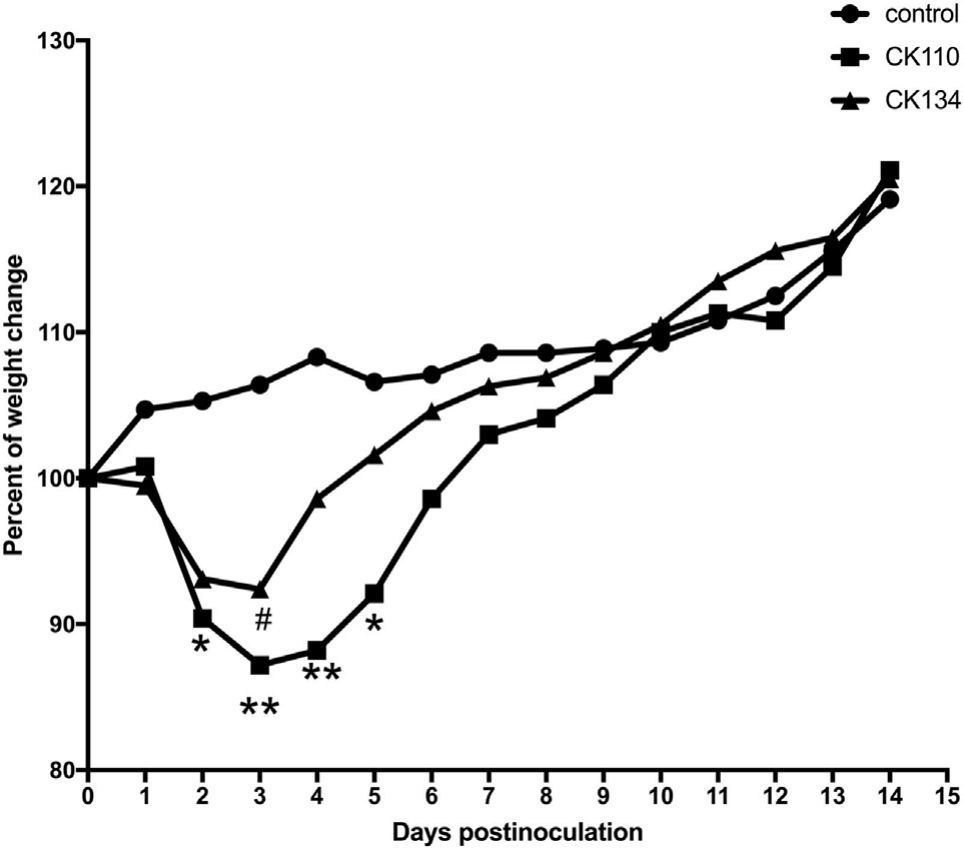
Weight changes in BALB/c mice for the 14 days postinoculation. Mice were inoculated intranasally with the H7N9 viruses in a volume of 0.05 ml of viral stock. Mice inoculated with phosphate-buffered saline served as a negative control group. Each value was the percent change in the initial mean starting weight on day 0 (21). Statistical analysis was performed using the two-way ANOVA test (GraphPad Prism 7.0 software), *p* < 0.05 was considered statistically significant (*p < 0.05; **p < 0.01).

Statistical analyses were done using GraphPad Prism 7.0 software (GraphPad Software Inc., San Diego, CA, USA), The Student’s *t*-test was used in the Table 3 and two-way ANOVA test was used in the Figures 1 and 2. A *p*-value < 0.05 was considered to be statistically significant (*p < 0.05; **p < 0.01).

## Results

### Pathogenicity of H7N9 Viruses in Chickens

To investigate the pathogenicity of the H7N9 viruses in chickens, we inoculated chickens with 10^8^EID_50_ of CK134 or CK110 virus in a 0.2-ml volume. The inoculated and contacted chickens from the two experimental groups and the chickens from the control group were observed for clinical symptoms for at least 14 days.

None of the chickens showed any signs of disease, and all survived until the end of the experiment. All chickens in the inoculated group seroconverted at 14 DPI. At 3 DPI, CK110 and CK134 replicated to mean titers of 2.08 ± 0.63–2.33 ± 1.23, 1.58 ± 0.14–2.08 ± 1.01, and 1.58 ± 0.14–1.83 ± 0.38 log_10_EID_50_/ml in the lungs, brain, and trachea, respectively. Furthermore, CK134 could also replicate to a mean titer of 1.58 ± 0.14 log_10_EID_50_/ml in the intestines (Table 1). By 5 DPI, CK134 had replicated in multiple organs of the inoculated chickens and their mean titers were 2.17 ± 1.15, 2.50 ± 1.73, 1.83 ± 0.58, 3.58 ± 1.84, 2.50 ± 1.73, 1.92 ± 0.72, 2.17 ± 1.15, 1.83 ± 0.58, and 3.50 ± 1.73 log_10_EID_50_/ml in the heart, liver, spleen, lungs, kidneys, brain, intestines, pancreas, and trachea, respectively. CK110 was detected in most organs of the inoculated chickens except for the spleen and intestines; the mean titers were from 1.83 ± 0.58 to 3.67 ± 1.26 log_10_EID_50_/ml in these organs, respectively (Table 1). According to the results, the two H7N9 viruses isolated from poultry in Guangdong could be detected in the lungs, and one could be done in the intestines of the inoculated chickens. Therefore, we confirmed that some H7N9 viruses might replicate in the respiratory system and digestive system.

**Table 1:** Replication in chickens of the H7N9 viruses after intranasal inoculation.

Strains	Time (DPI)	Viral titers in organs (log_10_EID_50_/ml)^a^								
		Heart	Liver	Spleen	Lung	Kidney	Brain	Intestine	Pancreas	Trachea
CK134	3	ND^b^	ND	ND	2.33 ± 1.23	ND	2.08 ± 1.01	1.58 ± 0.14	ND	1.83 ± 0.38
	5	2.17 ± 1.15	2.5 ± 1.73	1.83 ± 0.58	3.58 ± 1.84	2.5 ± 1.73	1.92 ± 0.72	2.17 ± 1.15	1.83 ± 0.58	3.50 ± 1.73

CK110	3	ND	ND	ND	2.08 ± 0.63	ND	1.58 ± 0.14	ND	ND	1.58 ± 0.14
	5	2.17 ± 1.54	1.75 ± 0.43	ND	3.67 ± 1.26	2.75 ± 1.56	1.83 ± 0.58	ND	2.17 ± 1.54	3.50 ± 1.89

*^a^A value of 1.5 was assigned for calculations (21) if the virus was not detected from the undiluted sample in three embryonated hen eggs. Viral titers were expressed as mean ± SD in log_10_EID_50_/ml of tissue*.

*^b^Not detected*.

### Shedding of H7N9 Influenza Viruses in Chickens

To measure the viral shedding, oropharyngeal and cloacal swabs from the chickens inoculated with CK110 or CK134 virus were obtained on days 3, 5, 7, 9, 11, and 14 DPI. The results showed that the CK134 virus might have better replication capacity than the CK110 virus in chickens; therefore, oropharyngeal and cloacal swabs were collected from the chickens inoculated with the CK134 virus at 17, 20, 23, 25, 27, and 30 DPI in order to study for how long the viral shedding persists.

The viral shedding of the CK110-inoculated group was detected from oropharyngeal swabs of all of the inoculated chickens at 3, 5, 7, 9, 11, and 14 DPI and from cloacal swabs of 0/5 to 5/8 chickens at 3 to 14 DPI (Table 2). In the CK134-inoculated group, viral shedding was observed from the oropharyngeal swabs of 4/5–11/11 of chickens at 3, 5, 7, 9, 11, and 14 DPI and could still be detected from the oropharyngeal swabs of 4/5, 4/5, 3/5, 1/5, 1/5, and 1/5 chickens at 17, 20, 23, 25, 28, and 30 DPI, respectively. Viral shedding was detected from the cloacal swabs of 3/11 to 5/8 chickens from 3 to 14 DPI and the CK134 virus could also be detected from the cloacal swabs of 2/5 chickens at 17 DPI and 1/5 chicken at 20 DPI (Table 2). The results showed that chickens infected by the H7N9 viruses shed and maintained the virus for a long period of time. This was especially true for the CK134 virus, which could still be detected from oropharyngeal swabs at 30 DPI and from cloacal swabs until 20 DPI. Therefore, we concluded that these H7N9 avian influenza viruses shed well not only in the respiratory system but also in the digestive system, and the viral shedding of the inoculated chickens could be sustained for a long time.

**Table 2:** Viral shedding in cloacal and oropharyngeal swabs from inoculated and contacted chickens^a^.

Virus	Viral shedding of chickens inoculated intranasally with 10^8^EID_50_ of virus				Viral shedding of chickens contacted to virus-inoculated chickens		
	DPI^b^	Number shedding/total number		Sero conversion (positive/total)	Number shedding/total number		Sero conversion (positive/total)
		Oropharyngeal	Cloacal		Oropharyngeal	Cloacal	
CK134	3	11/11	3/11	5/5	5/5	0/5	5/5
	5	8/8	5/8		5/5	4/5	
	7	5/5	3/5		5/5	5/5	
	9	5/5	3/5		5/5	3/5	
	11	5/5	3/5		5/5	2/5	
	14	4/5	3/5		5/5	2/5	
	17	4/5	2/5		5/5	2/5	
	20	4/5	1/5		5/5	0/5	
	23	3/5	0/5		3/5	0/5	
	25	1/5	0/5		2/5	0/5	
	28	1/5	0/5		1/5	0/5	
	30	1/5	0/5		1/5	0/5	

CK110	3	11/11	4/11	5/5	3/5	1/5	5/5
	5	8/8	5/8		4/5	1/5	
	7	5/5	3/5		4/5	2/5	
	9	5/5	3/5		4/5	2/5	
	11	5/5	2/5		4/5	2/5	
	14	5/5	0/5		4/5	2/5	

*^a^The data showed the viral shedding in inoculated and contacted chickens that each chicken was numbered with a metal ring to the leg (21)*.

*^b^DPI represented days postinoculation. The contact chickens were placed in the same isolator to allow contact with the chickens inoculated with each virus for 24 h postinoculation*.

### Transmission of H7N9 Influenza Viruses in Chickens

To investigate the transmissibility of the CK134 and CK110 vi-ruses, five chickens were placed in each inoculated group to allow for contact with the inoculated chickens for 24 h postinoculation. Oropharyngeal and cloacal swabs of the naïve contact chickens were obtained at 3, 5, 7, 9, 11, and 14 DPI. Only swab samples from the naive chickens housed with the CK134-inoculated group were collected at 17, 20, 23, 25, 28, and 30 DPI.

During the observation period, no obvious clinical signs were seen in the contact chickens. All of the chickens in the two groups seroconverted at 14 DPI. CK110 viral shedding was detected from the oropharyngeal swabs of 3/5 contacted chickens at 3 DPI, and CK110 viral shedding was detected from oropharyngeal swabs of 4/5 to 4/5 of the contacted chickens at 5, 7, 9, 11, and 14 DPI. Viral shedding was detected from cloacal swabs of 1/5 to 2/5 of the contacted chickens at 3 to 14 DPI (Table 2).

For the CK134-contacted chickens, viral shedding could be detected in the oropharyngeal swabs of all five of the chickens at 3 to 20 DPI, and the virus could still be detected from the oropharyngeal swabs of 3/5, 2/5, 1/5, and 1/5 chicken at 23, 25, 28, and 30 DPI, respectively. The virus could not be detected from the cloacal swabs at 3 DPI. 4/5 chickens showed viral shedding in the cloacal swabs at 5 DPI. Viral shedding could be detected from the cloacal swabs in all chickens at 7 DPI. From 9 to 17 DPI, viral shedding could be detected from the cloacal swabs 2/5 to 3/5 chickens. From 20 to 30 DPI, the virus could not be detected from the cloacal swabs (Table 2).

The results showed that the cohabitating chickens could be infected by the H7N9 virus through contact, and the virus could be detected for a long time. The CK134 virus could be detected from the oropharyngeal swabs and cloacal swabs until 30 and 17 DPI, respectively. Therefore, our results indicated that the H7N9 viruses could be transmitted through the respiratory system and the digestive system of the inoculated chickens to contacted chickens and that the viral shedding of the contacted chickens could last for a long time.

### Serological Analysis of Serum Samples From Chickens

We next examined the humoral immune response and its duration in inoculated chickens and naïve contact chickens. To this end, serum samples were collected from the CK134-inoculated and naïve contact chickens at 9, 11, 14, 17, 21, 25, 28, and 30 DPI. All of the serum samples were analyzed by HI test.

In the CK134-inoculated group, the average HI antibody titers were 2^5^ (*p* > 0.05), 2^5.2^ (*p* > 0.05), 2^6.4^ (*p* < 0.01), 2^6.6^ (*p* < 0.01), and 2^7.2^ (*p* < 0.01) at 9, 11, 14, 17, and 21, respectively. The mean HI antibody titers reached 2^7.3^ (*p* < 0.01), 2^7.5^ (*p* < 0.01), and 2^7.3^ (*p* < 0.01) at 25, 28, and 30 DPI (Figure 1).

For the chickens in contact with the CK134-inoculated group, we also collected serum samples at 9, 11, 14, 17, 21, 25, 28, and 30 DPI. HI antibody could be also detected in these contact chickens at 9 and 11 DPI, and the mean HI antibody titers were 2^5.6^ (*p* > 0.05) and 2^6.0^ (*p* > 0.05). The mean HI antibody levels increased dramatically over the next few days, and the average HI antibody titers of these contact chickens were 2^6.6^ (*p* < 0.01) and 2^6.8^ (*p* < 0.01) at 14 and 17 DPI. From 21 to 28 DPI, the contact chickens maintained mean HI antibody titers of 2^6.8^ (*p* < 0.01). At the end of the experiment, the mean HI antibody levels reached 2^7.2^ (*p* < 0.01) (Figure 1).

Overall, antibodies were detected in all of the chickens infected with the CK134 virus for up to 30 DPI, and they had relatively high serum HI titers. For the naïve contact chickens housed with chickens infected by the CK134 virus, the antibody levels were consistently high. These serology data demonstrated that the H7N9 avian influenza viruses replicate well in chickens and relatively high antibody levels likely protect the host well against disease.

### Expression of PRR and Cytokines in Lungs and Brain of Chickens

To determine the immune responses of chickens inoculated with the H7N9 virus, the expression of PRRs and cytokines were detected with qRT-PCR in the lungs and brain of the inoculated chickens and control chickens at 3 DPI. In the brain, the CK134 virus induced the upregulation of membrane-bound receptors (TLR3, TLR7, MDA5), cytokines (IL-1β, IL-6, IL-8, TNFα, IFN-α, IFN-β, IFN-γ), and antiviral molecules (PKR and Mx) compared to the control chickens. The mRNA levels of TLR3, TLR7, and MDA5 increased approximately 9-fold (*p* < 0.01), 8-fold (*p* < 0.01), and 6-fold (*p* < 0.01), respectively. The CK134 virus increased the expressions of IL-1β, IL-6, and IL-8 by approximately 4-fold (*p* < 0.05), 7-fold (*p* < 0.01), and 6-fold (*p* < 0.01), respectively. TNFα expression increased 4-fold (*p* < 0.05) compared to the control chickens. The expressions of the IFN-α, IFN-β, and IFN-γ increased approximately 20-fold (*p* < 0.01), 11-fold (*p* < 0.01), and 9-fold (*p* < 0.01), respectively. The induction of PKR and Mx mRNA increased by 4-fold (*p* < 0.05) and 17-fold (*p* < 0.01), respectively (Table 3).

**Table 3:** Pattern-recognition receptors (PRRs) and cytokine expression in inoculated chickens after infection with the H7N9 viruses^a^^,^^b^.

Gene^c^	Relative expression mRNA level over control in organs (mean ± SD)	
	Brain	Lungs
TLR3	9.48 ± 1.61**	1.89 ± 0.04**
TLR7	8.33 ± 1.30**	1.60 ± 0.06**
MDA5	6.44 ± 0.59**	1.22 ± 0.13*
PKR	3.84 ± 0.47**	1.09 ± 0.10
Mx	17.55 ± 0.35**	0.71 ± 0.04**
IL1β	3.55 ± 0.12**	0.53 ± 0.03**
IL6	7.80 ± 1.34**	1.51 ± 0.06**
IL8	6.14 ± 2.93*	1.14 ± 0.09
IFNα	20.17 ± 0.41**	1.60 ± 0.05**
IFNβ	11.79 ± 0.58**	1.18 ± 0.05**
IFNγ	9.46 ± 0.73**	2.32 ± 0.23**
TNFα	3.61 ± 0.09**	2.46 ± 0.96

*^a^The relative gene expression was calculated as follows: fold change = 2^–ΔΔ Ct,^ where Δ Ct = Ct target − Ct β-action, and ΔΔ Ct = ΔCt treated − ΔCt control. The result of the relative gene expression in control group was 1*.

^b^Statistical analysis was performed using the Student’s *t*-test, *p* < 0.05 was considered statistically significant (*p < 0.05; **p < 0.01).

*^c^The inspection items included the PRRs, antiviral molecules, and cytokines, which were detected with qRT-PCR in the lungs and brain of the inoculated chickens and control chickens at 3 DPI*.

In the lungs, the expressions of PKR, IL-8, and IFN-β mRNA were almost equal to those of the control chickens (1.1-fold, *p* > 0.05; 1.1-fold, *p* > 0.05; and 1.2-fold, *p* > 0.05, respectively). The expressions of Mx and IL-1β in the inoculated chickens were lower than in the control chickens (0.7-fold induction for Mx (*p* < 0.01), and 0.5-fold induction for IL-1β (*p* < 0.01)) with elevated levels of, TLR3, TLR7, MDA5, IL-6, IFN-α, and IFN-γ. The expressions of TLR3, TLR7, MDA5, IL-6, IFN-α, and IFN-γ increased by approximately 2-fold (*p* < 0.01), 1.6-fold (*p* < 0.01), 1.2-fold (*p* < 0.05), 1.5-fold (*p* < 0.01), 1.5-fold (*p* < 0.01), and 2.3-fold (*p* < 0.01), respectively (Table 3).

According to the result of statistical analysis performed with the Student’s *t*-test, in the inoculated chickens, the overall expression levels of the PRRs and cytokines were upregulated in the brain, and the expressions of TLR3, TLR7, MDA5, IL-6, IFN-α, and IFN-γ also increased in the lungs.

### Pathogenicity of H7N9 Viruses in Mice

To detect pathogenicity in mice, female SPF BALB/c mice were inoculated with CK110 and CK134 viruses at doses of a 0.05 ml of the stocks solution. Body weight, disease symptoms, and mor-tality were monitored daily for 14 days. All mice exposed to the CK110 and CK134 viruses survived to 14 DPI and no obvious signs of disease were present. However, mice inoculated with either of these two H7N9 viruses did experience weight loss. Compared to the mice in the negative control group, CK110 caused about 10% (*p* < 0.05), 13% (*p* < 0.01), 12% (*p* < 0.01), and 8% (*p* < 0.05) body weight loss at 2, 3, 4, and 5 DPI (Figure 2), respectively. CK134 caused around 8% (*p* < 0.05) body weight loss at 3 DPI (Figure 2).

To evaluate the replication of these H7N9 viruses in mice, the brain, spleen, kidneys, and lungs of inoculated and negative control mice were collected at 3 and 5 DPI for virus replication, respectively. Compared to the mice in the negative control group, CK110 and CK134 were detected only in the lungs at 3 DPI and replicated to mean titers of 2.92 ± 1.01 and 2.08 ± 0.14 log_10_EID_50_/ml, respectively (Table 4); at 5 DPI, both viruses were de-tected only in the lungs and replicated to mean titers of 2.08 ± 1.13 and 2.00 ± 0 log_10_EID_50_/ml, respectively (Table 4).

**Table 4:** Replication of the H7N9 viruses in mice after intranasal inoculation.

Strains	Time (DPI)	Viral titers in organs (log_10_EID_50_/ml)^a^			
		Spleen	Lungs	Kidneys	Brain
CK134	3	ND^b^	2.08 ± 0.14	ND	ND
	5	ND	2.00 ± 0	ND	ND

CK110	3	ND	2.92 ± 1.01	ND	ND
	5	ND	2.08 ± 1.13	ND	ND

*^a^A value of 1 was assigned for calculations (21) if the virus was not detected from the undiluted sample in three embryonated hen eggs. Viral titers were expressed as mean ± SD in log_10_EID_50_/ml of tissue*.

*^b^Not detected*.

Overall, the pathogenicity of these H7N9 viruses isolated from poultry showed mild results in mice.

## Discussion

Previous studies on the tissue tropism of H7N9 viruses isolated from humans in chickens have produced varied results. Ku et al. found that the H7N9 virus could be detected in the trachea of infected chickens for only 2 DPI and in the lungs for 3 and 5 DPI (14). Pantin-Jackwood et al. demonstrated that low viral titers (10^0.97^ to 10^1.23^ EID_50_) of the H7N9 virus were detected in the intestines and spleen of chickens at 3DPI, but not in the lungs and kidneys (12). Kalthoff et al. showed the replication of the H7N9 virus could be detected in intestines of infected chickens, but not in the lungs (11). We found that these H7N9 viruses could replicate well in the upper and the lower respiratory tracts, the mean titer in the trachea reached 3.50 log_10_EID_50_/ml, and mean titer in the lungs reached 2.33 log_10_EID_50_/ml at 3 DPI and 3.67 log_10_EID_50_/ml at 5 DPI. Furthermore, our isolates were also able to spread to the central nervous system, heart, liver, spleen, kidneys, pancreas, and intestines.

Studies have demonstrated that the few amino acid changes detected in human cases of H7N9 might be associated with adaptation and virulence. Genetic analysis of the H7N9 viruses in our study showed that the two viruses both possessed a single basic amino acid at the cleavage site of HA (EIPKGR*GL; *indicated the cleavage site), and T160A, G186V, S199N, and Q226L substitutions (H3 numbering) were identified at the receptor-binding site of hemagglutinin. The two H7N9 viruses encoded a deletion at positions 69–73 (N9 numbering) of the NA stalk region similar to the H7N9 human isolates. Signature mutations in other proteins were also identified in our viruses, such as L89V in PB2, and V100A, K365R, and S409N in PA; N30D and T215A in M1; and P42S in NS1. However, the mutations E627K and D701N mutations in PB2 that were usually associated with the adaptation and virulence of the avian influenza virus were not found in our viruses (23).

We suspected that one reason for the difference in replication ability of H7N9 in chickens might be the different origins of the isolates. Our viruses were isolated from chickens in live-poultry markets, which made them more suitable for replicating in chickens. Because they lack a polybasic cleavage site, LPAI viruses are thought to be secreted only by cells of the respiratory and intestinal tract. However, a limited number of chicken-isolated LPAI strains have been isolated from other tissues beyond the respiratory and intestinal tract, including the pancreas, kidneys, and oviduct in inoculated chickens (24). Another reason might be the use of different virus inoculation routes and challenge doses (23). However, the pathogenic mechanism of avian influenza virus is complex and the role of other mutations and factors in H7N9 pathogenicity cannot be ruled out. Therefore, more re-search on the pathogenic mechanisms of the H7N9 virus should be carried out in the future.

Generally, the routes of infection and dissemination of avian influenza viruses include respiratory and olfactory routes, which may be affected by the number of viral particles trapped in mucous secretions in the respiratory and digestive tracts. Zhang et al. observed that chickens were easily infected with H7N9 viruses and efficiently shed the virus for up to 7 days (13). Kalthoff et al. demonstrated that chickens infected with the H7N9 virus shed large viral RNA loads from 2 to 10 DPI in the respiratory swabs and until 14 DPI in cloacal swabs (11). In our study, in chickens inoculated with CK110, the virus was detected from oropharyngeal swabs for up to 14 DPI and from cloacal swabs for up to 11 DPI. In chickens inoculated with CK134, the virus could be detected from oropharyngeal swabs until 30 DPI, and from cloacal swabs for up to 20 DPI. According to these results, the shedding of H7N9 LPAI virus in chickens infected through the respiratory and digestive tracts could last for a long time in living poultry, which may cause a reciprocating cycle of infection among chickens.

The long-term shedding of H7N9 viruses in infected chickens through the respiratory and digestive tracts may lead to the transmission and spread among neighboring animals. Previous studies demonstrated that many types of animals (chickens, ferrets, guinea pigs, and pigs) carrying H7N9 viruses could infect other animals by droplets and contact. However, Ku et al. found that the H7N9 virus could not be transmitted efficiently from infected chickens to naïve chickens (14). In our study, CK110 viral shedding was detected from oropharyngeal swabs in 3/5 to 4/5 of the contacted-chickens and from cloacal swabs in 1/5 to 2/5 of contacted-chickens from 3 to 14 DPI. For the CK134-contacted chickens, viral shedding could be detected in the oropharyngeal swabs of all five of the chickens at 3 to 20 DPI, and 1/5 to 3/5 of the chickens from 23 to 30 DPI. Furthermore, in 2/5 to 5/5 of the chickens, the virus was detected from the cloacal swabs from 5 to 17 DPI. Our results confirmed that the H7N9 virus was effectively transmitted among chickens through contact, and the transmission could last for a long time. However, we could not exclude that the chickens could have suffered a reciprocating cycle of infection during the experiment.

Previous phylogenetic analyses revealed that the H7N9 influenza virus outbreak in China in 2013 was a reassortment. Although the precise source of the HA and NA segments of the H7N9 virus could not be determined, there is a consensus that the six internal gene segments of the H7N9 virus originated from the H9N2 avian influenza viruses (23). Interestingly, surveillance of the H9N2 avian influenza virus in Eastern China has revealed that most viruses before 2000 were capable of propagating in inoculated chickens but were not transmissible through respiratory droplets. However, after 2001, the H9N2 viruses not only replicated well *in vivo* but were also transmitted efficiently by respiratory droplets in chickens. More interestingly, the H9N2 viruses isolated during 2010–2013 showed high isolation rate and titers, as well as longer periods of viral shedding, especially from the cloaca in challenged chickens (25). Thus, we suspected that the internal gene segments of the H7N9 virus originating from the H9N2 viruses might play a big role in transmission and shedding. Therefore, when humans are exposed to chickens that have been carrying the H7N9 virus for a long time, the risk of infection will be greatly increased, especially among salesmen and butchers.

Influenza virus infection can directly activate both the innate and adaptive immune responses in animals. The innate immune system is the first line of defense against influenza virus infection and initiates the recognition of pathogen-associated molecular patterns by PRRs, which trigger antiviral signaling cascades that result in the production of interferons (IFNs), cytokines, and che-mokines (26). Research has examined the host cytokine and chemokine profiles in the serum and lungs of H7N9 patients and the results showed that high levels of cytokines and chemokines were partially responsible for the disease progression (27, 28). Mice infected with the H7N9 virus produced high levels of pro-inflammatory cytokines in their lungs and serum (29). Ku et al. showed that the toll-like receptors (TLR1, 2, 3, 4, 5, 7, and15) and inflammatory cytokines (TGF-β3, TNFα, IFN-α, IFN-β, IL-1β, IL-2, IL-4, IL-8, and IL-10) in the lungs of chickens infected with the H7N9 virus had no significant changes at 3 DPI (14). In our study, we also found that the induction of PRRs, cytokines, and antiviral molecules occurred in the lungs and brain of chickens infected with the H7N9 virus. However, the levels of induction for the PRRs (TLR3, TLR7, and MDA5), cytokines (TNFα, IFNα, IFNβ, IFNγ, and IL-6), and antiviral molecules (PKR and Mx) showed little change compared to the control chickens at 3 DPI. In contrast to the lungs, the expressions of these molecules in the brain of chickens infected with the H7N9 virus were significantly upregulated compared to the control chickens at 3 DPI. Our results suggested that the diversity of the immune response and cytokines in different organs might be related to the tissue tropism of the virus. Further studies should explore this hypothesis in the future.

The adaptive immune response is activated after the innate immune response (30). The host cells generate virus-specific antibodies to defend against viral antigens (31). Pantin-Jackwood et al. found that most chickens infected with the H7N9 virus had detectable titers of antibodies against the virus at 11 DPI (12). In our study, we found that all of the chickens inoculated with the CK134 virus had relatively high serum HI titers at 30 DPI, and the HI titers could reach up to 2^7.5^. Naïve contact chickens housed with chickens infected with the CK134 virus could also develop high antibody levels at 30 DPI, and the highest mean HI titers could reach 2^7.2^. Previous studies demonstrated that the hospitalized H7N9 patients who survived had elevated levels of antibodies compared to those who died and therefore speculated that the presence of antibodies might improve clinical outcomes in infected patients (16). Our results seemed to confirm that relatively high antibody levels might protect chickens from the disease, thereby resulting in reduced morbidity and mortality. Chickens could only carry the H7N9 virus and not show any clinical symptoms when the pathogenicity of the virus and immune response of the host reached equilibrium.

Mice are usually used as the primary model for evaluating the pathogenicity of the influenza virus in mammals. The H7N9 viruses isolated from humans can be detected in the turbinate, lungs, brain, and spleen of mice (32, 33), However, H7N9 viruses isolated from poultry only replicated in the lungs of mice (13). In our study, the two H7N9 viruses isolated from poultry could replicate only in the lungs of mice. By the end of 14 DPI, mice inoculated with the H7N9 viruses had no signs of illness or death, but they experienced weight loss. Therefore, the pathogenesis is likely different between H7N9 viruses isolated from humans and poultry.

## Ethics Statement

All experiments were carried out in ABSL-3 facilities in compliance with approved protocols (SCAUABSL2015-0011) by the Biosafety Committee of South China Agriculture University. All animals were handled in accordance with the approved guidelines of the Experimental Animal Administration and Ethics Committee of South China Agriculture University approved guideline.

## Author Contributions

PJ designed this study and performed the experiments. YS participated in the data collection and analysis. JH, CX, JC, SW, NQ, NW, and GO assisted with animal experiment. PJ and YS drafted the manuscript. ML participated in writing the discussion. All authors have read and approved the final manuscript.

## Conflict of Interest Statement

The authors declared that the research was conducted in the absence of any commercial or financial relationships that could be construed as a potential conflict of interest.
